# The Effect of Breast Cancer and Breast Self‐Examination Health Education on Awareness, Beliefs, and Practices of Female Students at the Faculty of Health Sciences of a University in Somalia

**DOI:** 10.1155/tbj/8854280

**Published:** 2026-03-26

**Authors:** Şeyma Zehra Altunkurek, Eylül Yeşilyurt, Samira Hassan Mohamed

**Affiliations:** ^1^ Department of Nursing, Somalia Mogadishu Recep Tayyip Erdoğan Faculty of Health Sciences, University of Health Sciences, Mogadishu, Somalia, uhs.edu.kh; ^2^ Department of Nursing, Gulhane Health Sciences Institute, University of Health Sciences, Ankara, Türkiye, akdeniz.edu.tr

**Keywords:** awareness, belief, breast cancer, breast self-examination, health education, Somalia, students

## Abstract

**Background and Aims:**

Breast cancer is the most common malignancy among women globally, and early detection remains the cornerstone of reducing mortality. In low‐resource settings such as Somalia, limited access to mammography and diagnostic services makes breast self‐examination (BSE) an important awareness practice. This study aimed to assess the effect of an educational intervention based on the health belief model (HBM) on Somali female university students’ knowledge, beliefs, and practices related to breast cancer and BSE.

**Methods:**

A randomized controlled study was conducted among 86 female undergraduate students enrolled at the Faculty of Health Sciences, University of Health Sciences, Mogadishu, Somalia, between September 2021 and June 2022. Participants were randomly assigned to an intervention group (*n* = 43) or a control group (*n* = 43) using the sealed opaque envelope method. Data were collected through a structured questionnaire including a sociodemographic form and the Champion’s Health Belief Model Scale (CHBMS). The intervention group attended a structured 90‐min HBM‐based breast health education session delivered by two public health lecturers, while the control group received no education during the study period. Three months after the intervention, posttest data were collected from both groups. Statistical analyses were performed using descriptive and inferential tests, with significance set at *p* < 0.05 (two‐tailed).

**Results:**

The mean age of participants was 20.3 ± 1.63 years. Postintervention, the intervention group showed significant improvements in knowledge of breast cancer and BSE (*p* < 0.001), perceived benefits (*p* = 0.02), health motivation, and self‐confidence in performing BSE (*p* < 0.001). The frequency and correct practice of BSE also increased significantly after 3 months (*p* < 0.001).

**Conclusion:**

HBM‐based breast health education improved students’ knowledge, awareness, and BSE practice. Although BSE is not a screening tool, it is an essential awareness method in settings with limited access to diagnostic services.

## 1. Introduction

Breast cancer is the most common cancer in women worldwide. According to the World Health Organization (WHO), 2.3 million women were diagnosed with breast cancer in 2022, resulting in 685,000 deaths worldwide [1]. According to the WHO report “Assessment of Breast Cancer Control Capacities in the WHO African Region (2022),” approximately 146,130 new breast cancer cases and 71,662 breast cancer–related deaths are estimated in sub‐Saharan Africa in 2022. Research indicates that regions, such as South Asia, Central, and sub‐Saharan Africa, have high mortality rates due to limited healthcare infrastructure, late‐stage diagnosis, limited screening and treatment resources, and inadequate access to treatment [2, 3]. According to the World Cancer Observatory data, breast cancer is the most common and deadly type of cancer in Somalia. Globocan 2022 data reported 1811 new cases and 1165 deaths in Somalia [4, 5].

Early diagnosis and intervention can significantly reduce morbidity and mortality rates related to breast cancer and facilitate a more successful treatment process. The American Cancer Society (ACS) recommends mammography, clinical breast examination (CBE), and breast self‐examination (BSE) for early detection of breast cancer [6]. Mammography is one of the most effective and widely used imaging methods for early diagnosis. Recent studies have shown that mammography screening significantly reduces mortality rates [7–10]. The WHO recommends regular mammography screening for women aged 40–75 [9]. In Somalia, this screening method is not widely used due to limited access to healthcare, the high cost of mammography, and the scarcity of mammography devices. A sufficient number of qualified healthcare workers are needed to perform mammography. While its use in Somalia is not impossible, it is not effectively used due to these factors [4, 11–13]. Due to the scarcity of mammography in Somalia, BSE is an important method for examination. While the WHO and ACS guidelines do not recommend BSE as a screening method, it is considered an awareness‐raising tool in low‐resource countries [6, 14].

In the “Guidelines for the early detection and screening of breast cancer” prepared by the WHO for the Eastern Mediterranean Region (EMRO), it is stated that BSE can be emphasized in breast health education [14]. Because breast lumps are often self‐diagnosed, BSE is important. BSE is a simple, low‐cost, and safe method that helps women become familiar with their bodies and detect unusual changes early. No special equipment is required when performing a BSE [14]. The procedure takes approximately five minutes and is recommended for all women aged 20 and older [14]. However, for BSE to be effective, sufficient knowledge and proper application techniques must be acquired. Promoting BSE among all women from an early age and ensuring its regular practice is considered a crucial nursing responsibility for the early detection of cancer [15, 16]. It is crucial for university students, in particular, to understand the importance of BSE and to practice it regularly in the coming years. Therefore, the use of theoretical models that will support students in adopting early diagnosis behaviors is important.

Various models are used to help individuals adopt positive health behaviors. The health belief model (HBM) is one of the most frequently used models that can be effective in instilling positive health behaviors in individuals during breast cancer screening and in examining cancer prevention behaviors [13, 17]. The literature contains studies on its use in health education and promotion, as well as in maintaining and changing health status [13, 15, 17, 18]. The HBM encompasses aspects such as perceived susceptibility to the disease, perceived severity, perceived barriers, perceived benefits, self‐efficacy, and cues to action. It is important to emphasize the impact of barriers affecting behavior change [18]. Researchers have recently observed that women’s health views have an impact on their early detection practices for breast cancer [13]. This theoretical framework clarifies the connection between a person’s beliefs and actions, as well as the factors that shape a person’s health‐related behaviors and inspire them to action [13, 15]. The HBM states that women who are at risk for breast cancer are more likely to get the disease if they view it as a serious condition, have a low perception of incapacity, and have a high perception of benefit [13]. The HBM served as the foundation for the development of breast cancer and BSE health education in this study.

In the literature, awareness of BSE among university students has generally been found to be insufficient (Türkiye, Malaysia, Bangladesh, Nigeria, etc.) [15, 19–23]. However, previous studies generally used a single‐group pretest–posttest design. Education or discussion session‐based interventions in Malaysia, Bangladesh, India, and Türkiye have significantly increased students’ breast cancer knowledge and BSE practice rates [19, 22–24]. Therefore, it is necessary to increase students’ breast cancer awareness, encourage BSE, and emphasize the effectiveness of educational programs [16, 17, 25]. In a cross‐sectional study conducted by Altunkürek and Hassan among young women in Somalia, it was determined that women had insufficient knowledge about breast cancer and BSE, and that BSE practice rates were quite low [13]. However, there are no randomized controlled trials evaluating the effectiveness of BSE training in Somalia. To fill this gap in the literature, this study was planned.

This study aims to determine the effects of HBM‐based health education on knowledge, attitudes, health beliefs, and BSE behaviors about breast cancer and BSE among female students at a university in Mogadishu, Somalia.

## 2. Materials and Methods

### 2.1. Study Design

A single‐blind, randomized, controlled pretest–posttest experimental design was used for this research. Clinical trials were recorded (NCT06706141).

### 2.2. Participants and Setting

The study was conducted at the Recep Tayyip Erdoğan Faculty of Health Sciences in Mogadishu, Somalia, between September 2021 and June 2022. The population of the research consisted of 500 female students over the age of 18 studying at the Recep Tayyip Erdoğan Faculty of Health Sciences in Mogadishu, Somalia. A randomized controlled study was undertaken on 86 female undergraduate students.

### 2.3. Sample Size Estimation and Sampling Technique

The number of samples to be included in the experimental and control groups of the research was determined by power analysis. To detect a significant group effect, the minimum sample size was calculated as 80 students in total, with 40 students in each group, using a 95% confidence interval, 0.05 alpha margin of error, 0.80 research power, and a slightly above‐medium effect size of 0.64 (Cohen’s *d* ≥ 0.5) [26]. The sample size was calculated using the G∗Power 3.1.9.7 program. The number of students in the faculty varies between 50 and 53. Considering that there may be losses in the research, all students in the class were included; 50 women were included in the experimental group, and 53 women were included in the control group. However, since a total of 17 female students, 7 students in the experimental group and 10 students in the control group did not want to participate in the study and were under 18 years of age, the posttest data were completed with 86 female students, 43 in the experimental group and 43 in the control group.

#### 2.3.1. Inclusion and Exclusion Criteria

The inclusion criteria for this study consisted of female undergraduate students enrolled in the Faculty of Health Sciences at the University of Health Sciences, Mogadishu Recep Tayyip Erdoğan, Somalia, who were in the preparatory or first academic year, had sufficient proficiency in Turkish to understand the training content, were over 18 years of age, and voluntarily agreed to participate in the research. Students who were in their second, third, or fourth years of study, had prior formal education or extensive knowledge regarding breast cancer or BSE, were under the age of 18, or did not provide voluntary consent were excluded from participation.

### 2.4. Randomization Procedure and Participant Assignment

**FIGURE 1:**
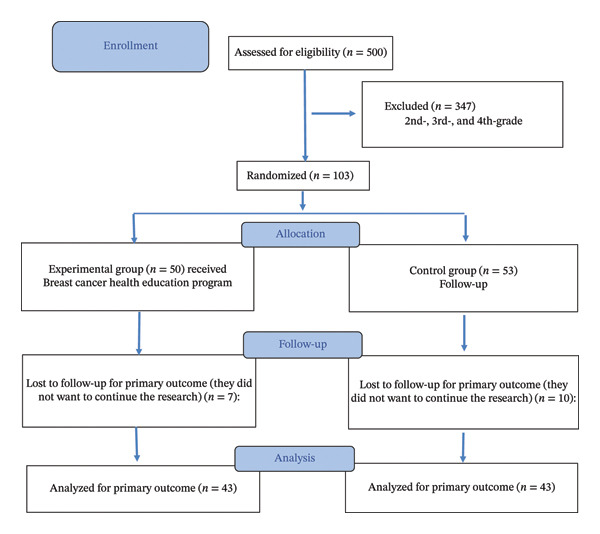
Flow diagram of study.

This study employed a single‐blind randomized controlled design to ensure methodological rigor and minimize selection bias. In this study, participants were not informed about whether they belonged to the intervention or control group to avoid potential bias in their responses and behaviors. However, the researchers who conducted the training were aware of the group assignments. This design ensured that participants remained blinded while maintaining the integrity of the intervention process. Randomization was carried out at the class level to prevent potential contamination between participants. Two classes within the Faculty of Health Sciences were identified as eligible units for inclusion. Participants were randomly assigned to the intervention and control groups by the sealed opaque envelope method [27]. Each class identifier was placed into identical envelopes, which were then randomly selected by an independent researcher who was not involved in participant recruitment, intervention delivery, or data analysis. The first class drawn was assigned to the intervention group, and the second to the control group, thereby maintaining allocation concealment and objectivity in the process. The intervention group participated in a structured HBM‐based breast cancer and BSE education program delivered through interactive presentations, demonstrations on breast models, and guided discussions. The control group did not receive any breast cancer or BSE education during the study period. They continued their usual academic schedule without any intervention. After the posttest data collection was completed, the same health education program provided to the experimental group was also offered to the control group for ethical reasons. A total of 86 students completed the study (43 in each group). The sequence of participant recruitment, randomization, follow‐up, and analysis is summarized in the CONSORT flow diagram (Figure 1).

### 2.5. Procedure and Intervention

This study employed a structured educational intervention based on the HBM to improve participants’ knowledge, beliefs, and practices regarding breast cancer and BSE. To ensure methodological consistency and reliability, the intervention was designed and delivered by two lecturers specializing in Public Health—one Turkish and one Somali—both serving as faculty members at the University of Health Sciences, Mogadishu, Somalia. Each had professional experience in community health education and nursing instruction. Prior to the implementation, both lecturers participated in a preparatory meeting to standardize the content, ensure fidelity to the intervention protocol, and maintain consistency across all training sessions. The intervention process consisted of three main stages: pretest data collection, HBM‐based educational training, and posttest follow‐up.

The experimental group received various interventions such as health education based on the HBM, individual counseling, and brochures on mammography screening. The training was given by two study team members in the faculty’s conference hall, in the form of a presentation, discussion, and question‐and‐answer sessions, with the experimental group divided into two groups, lasting approximately 90 min. It was completed in three sessions with breaks every 30 min. For training hours, free hours when students did not have classes were chosen. For each training group, a member of the study team first gave a presentation on breast cancer and breast screening behaviors and BSE (30 min). Then, a film was shown on the BSE practice recommended by the WHO (20 min) and training was given on how to perform correct and effective BSE on the breast model (20 min). A life‐sized silicone breast model (GPI Anatomicals, USA) was used to demonstrate and practice the correct BSE technique.

The training included topics such as breast anatomy; breast cancer incidence in Somalia, mortality, morbidity rates; breast cancer risk factors, importance of screening methods; the messages on BSE, HBMs, breast cancer awareness, confidence for BSE, and perceived barriers/benefits for BSE were presented in a PowerPoint presentation. The procedure for performing BSE was described using the English edition of Bristol Myers Squibb Oncology. In the movie, BSE was made simpler with schematics showing the breast area that needed to be covered. Simple directions on which fingers to use and how to move them over the breast were provided. After the film, the emphasis shifted to helping students gain more self‐assurance in their ability to accurately complete each BSE step. Students were encouraged to use proper palpation technique to find lumps by performing a breast examination on a silicone breast model. Feedback was given by the research team for each step. The training was completed by ensuring that each student performed BSE correctly. After the training, a brochure containing the BSE steps was given.

The control group did not receive any breast cancer or BSE education during the study period. They continued their usual academic schedule without any intervention. After the posttest data collection was completed, the same health education program provided to the experimental group was also offered to the control group for ethical reasons.

Data were collected at two time points: before the educational intervention (pretest) and 3 months after the intervention (posttest). The 3‐month interval was selected to allow sufficient time for participants to apply BSE practices and to assess the persistence of educational effects.

### 2.6. Instrument of Data Collection

#### 2.6.1. Sociodemographic Questionnaire

This survey included demographic questions, including the age of the students, the education level of the mother and father, family income level, and family history of breast cancer. There were also 7 questions about students’ breast cancer and BSE.

#### 2.6.2. Champion’s Health Belief Model Scale (CHBMS)

The 42‐item CHBMS [28] was refined in subsequent research (1993, 1997, and 1999) and was created by Victoria Champion in 1984 to assess women’s attitudes and beliefs regarding breast cancer and BSE [29]. Turkish translation of the scale was done by Karayurt and Dramalı [30]. In this study, the scale’s Turkish version was employed. CHBMS has six subscales. Perceived susceptibility (3 items) measures individuals’ perceived likelihood of developing breast cancer, while perceived severity (7 items) evaluates the level of concern regarding the seriousness and potential consequences of the disease. Perceived benefits (4 items) explore the extent to which participants recognize the advantages of performing BSE, and perceived barriers (11 items) identify the factors that may prevent or discourage regular BSE practice. Self‐Efficacy (10 items) assesses participants’ confidence in their ability to detect unusual breast changes through BSE, whereas Health Motivation (7 items) reflects general interest and responsibility toward maintaining personal health. Each subdimension of the scale is evaluated separately; the total score that includes the entire scale is not calculated. Women who score low on the “perception of barriers” subscale and score high on other subscales indicate that they have positive attitudes and beliefs about breast cancer and BSE practices [30]. The reliability and validity study of the original scale was conducted by Champion [29]. The internal consistency of Cronbach’s alpha coefficient of the scale was found to be between 0.69 and 0.90 for the subdimensions (Champion, 1993; 1997; 1999). In the Turkish adaptation of the scale, Cronbach’s alpha reliability coefficients were found to be between 0.58 and 0.89 for the subscales. In this study, Cronbach’s alpha coefficients were 0.68 for the perceived susceptibility subscale, 0.76 for perceived severity, 0.75 for perceived benefits, 0.67 for perceived barriers, 0.81 for self‐efficacy, and 0.84 for the health motivation subscale, indicating acceptable internal consistency across all subscales and supporting the reliability of the instrument for the study population.

### 2.7. Ethical Approval and Participant Consent

Ethical approval for this research was obtained from the Ethics Committee of the Mogadishu Somali‐Türkiye Recep Tayyip Erdoğan Training and Research Hospital (Approval No: MSTH/5090; Date: December 21, 2020). In addition, official authorization was received from the Faculty of Health Sciences, University of Health Sciences, Mogadishu, Somalia, before the commencement of data collection. Prior to participation, each student was thoroughly informed about the objectives, procedures, potential benefits, and minimal risks of the study. Both verbal and written information was provided, and voluntary written consent was obtained from all participants. They were assured that participation was entirely voluntary and that they could withdraw from the study at any time without penalty or explanation. To ensure confidentiality, no identifiable personal information was recorded in the data files. All research materials were securely stored with access limited exclusively to the research team. The study adhered to the ethical principles of the Declaration of Helsinki (2013 revision) and followed internationally accepted standards of scientific integrity, participant autonomy, and respect for human dignity.

### 2.8. Analysis of Data

Statistical analyses were performed using the Statistical Package for the Social Sciences (SPSS, Version 26.0; IBM Corp., Armonk, NY, USA). Descriptive statistics, including means, standard deviations, frequencies, and percentages, were calculated. The paired‐sample *t*‐test and independent‐sample *t*‐test were used for comparisons of normally distributed data, while the Wilcoxon signed‐rank test and Mann–Whitney *U* test were applied for nonparametric variables. The chi‐square test was used to compare categorical data. The significance level was set at *p* < 0.05 (two‐tailed). All analyses were prespecified, and no subgroup analyses were conducted. The statistical reporting adhered to the Statistical Analyses and Methods in the Published Literature (SAMPL) guidelines [31, 32].

## 3. Results

There were 86 female Somali university students in the sample group, ages 18 to 22. The mean age is 20.3 ± 1.63 years. In the experimental group, 34.8% of the mothers and 56.8% of the fathers had completed high school. In the control group, 53.7% of the fathers and 27.9% of the mothers had a high school level education. The economic status of 67.4% of the experimental group and 74.4% of the control group was at a moderate level. The experimental group had no family history of breast cancer. One person in the control group had a family history of breast cancer (*p* > 0.05) (Table 1).

**TABLE 1:** Demographic characteristics of participants (*N* = 86).

Characteristics	Experimental group (*n* = 43)		Control group (*n* = 43)		Statistics/*p* value
	Range		Range		
	Mean ± SD		Mean ± SD		
Age (years)	18–22		18–21		
	19.6 ± 1.35		21.1 ± 1.55		
	*n*	%	*n*	%	
Father’s educational level					
None	0	0.0	0	0.0	8.89^∗^/0.064
Primary	0	0.0	2	4.9	
Secondary	10	27.0	9	22.0	
High school	21	56.8	22	53.7	
University	12	16.2	8	19.4	
Mother’s educational level					
None	0	0.0	2	4.7	2.16^∗^/0.539
Primary	5	11.6	16	37.2	
Secondary	15	34.8	10	20.3	
High school	15	34.8	12	27.9	
University	8	18.8	3	9.9	
Family income status					
Poor	11	25.6	6	14.0	2.12^∗^/0.347
Middle	29	67.4	32	74.4	
High	3	7.0	5	11.6	
Do you have any family history of breast cancer					
Yes	0	0.0	1	2.3	1.01^∗∗^/0.314
No	43	100	42	97.7	

*Note:* Mean age: 20.3 ± 1.63. *p* < 0.05.

^∗^Chi‐square.

^∗∗^Fisher’s exact test.

Regarding knowledge of breast cancer, BSE, and BSE examination techniques, there was no discernible difference between the experimental and control groups’ pretest results (*p* > 0.05) (Table 2). The experimental group (81.4%) stated that they had heard of breast cancer, while 67.4% of the control group stated that they had heard of breast cancer. A total of 86.0% of the experimental group and 97.7% of the control group reported having no knowledge about breast cancer. Additionally, almost all of the women were aware of the early diagnosis of breast cancer, and only one person from the experimental group was aware of the issue. Of the participants in the experimental group, 79.1% had not heard of BSE, 90.7% had no knowledge of it, and 97.7% did not know how to perform it. Only one person in the experimental group reported performing BSE. It was determined that 97.7% of the participants in the control group had not heard of BSE, 97.7% had no knowledge of BSE, and 100% did not know how to perform BSE. No one in the control group performed BSE (Table 2).

**TABLE 2:** Comparison of awareness and practice of groups regarding breast cancer and BSE at pretest (*N* = 86).

Characteristics	Experimental group (*n* = 43)		Control group (*n* = 43)		*p*	Statistics
	*n*	%	*n*	%		
Have you heard about breast cancer						
Yes	35	81.4	29	67.4	0.138^∗^	2.19^∗∗^
No	8	18.6	14	32.6		
Knowledge of breast cancer						
Yes	6	14.0	1	2.3	0.049^∗^	3.89^∗∗^
No	37	86.0	42	97.7		
Knowledge of early diagnosis methods for breast cancer						
Yes	1	2.3	0	0.0	0.314^∗^	1.01^∗∗^
No	42	97.7	43	100.0		
Have you heard about breast self‐examination						
Yes	9	20.9	1	2.3	0.007^∗^	7.24^∗∗^
No	34	79.1	42	97.7		
Do you have knowledge of breast self‐examination						
Yes	4	9.3	1	2.3	0.167^∗^	1.91^∗∗^
No	39	90.7	42	97.7		
Know how to perform breast self‐examination						
Yes	1	2.3	0	0.0	0.314^∗^	1.01^∗∗^
No	42	97.7	43	100.0		
Do you practice breast self‐examination						
Yes	1	2.3	0	0.0	0.314^∗^	1.01^∗∗^
No	42	97.7	43	100.0		

^∗^= *p* < 0.05.

^∗∗^Fisher’s exact.

When the posttest results of the experimental and control groups regarding breast cancer, BSE knowledge, and BSE examination practices were compared, the entire experimental group 100% (*n* = 43) showed that they had knowledge about breast cancer, 97.7% (*n* = 42) knew breast cancer early diagnosis methods, had knowledge about BSE examination, and knew how to do it (*p* < 0.001). In addition, 86.0% (*n* = 37) of the experimental group reported that they had BSE examinations (*p* < 0.001) (Table 3).

**TABLE 3:** Comparison of awareness and practice of groups regarding breast cancer and BSE at posttest (*N* = 86).

Characteristics	Experimental group (*n* = 43)		Control group (*n* = 43)		*p* value	Statistics
	*n*	%	*n*	%		
Have you heard about breast cancer						
Yes	43	100.0	42	97.7	0.315	1.01^∗∗^
No	0	0.0	1	2.3		
Knowledge of breast cancer						
Yes	43	100.0	21	48.8	< 0.0001^∗^	29.56^∗∗^
No	0	0.0	22	51.2		
Knowledge of early diagnosis methods for breast cancer						
Yes	42	97.7	8	18.6	< 0.0001^∗^	55.23^∗∗^
No	1	2.3	35	81.4		
Have you heard about breast self‐examination						
Yes	42	97.7	27	62.8	< 0.0001^∗^	16.49^∗∗^
No	1	2.3	16	37.2		
Do you have knowledge of breast self‐examination						
Yes	42	97.7	14	32.6	< 0.0001^∗^	40.13^∗∗^
No	1	2.3	29	67.4		
Know how to perform breast self‐examination						
Yes	42	97.7	7	16.3	< 0.0001^∗^	58.11^∗∗^
No	1	2.3	36	83.7		
Do you practice breast self‐examination						
Yes	37	86.0	7	16.3	< 0.0001^∗^	36.64^∗∗^
No	6	14.0	36	83.7		

^∗^= *p* < 0.001.

^∗∗^Chi‐square.

The CHBM scale pretest and posttest comparisons of the groups are presented in Table 4. When the scale scores between the experimental and control groups were compared after the intervention, a significant difference was found in the perceived BSE benefits, perceived barriers, perceived confidence/self‐efficacy of BSE, and perceived health motivation subscales (*p* > 0.05).

**TABLE 4:** Comparison of mean CHBM subscales of participants in the groups (*N* = 86).

Scale and its subdimensions		Group	(Pretest) mean ± SD	(Posttest) mean ± SD	*p* ∗
Subscales	Perceived susceptibility	Experimental	5.28 ± 2.21	7.07 ± 2.70	**0.0021**
		Control	5.98 ± 2.94	6.86 ± 2.57	0.1937
		*p* ^∗∗^	0.216	0.714	
	Perceived severity	Experimental	21.86 ± 5.24	20.35 ± 4.84	0.1640
		Control	19.77 ± 5.25	19.60 ± 6.37	0.8963
		*p* ^∗∗^	0.068	0.544	
	Perceived BSE benefits	Experimental	12.95 ± 4.31	14.91 ± 3.31	**0.02**
		Control	12.49 ± 4.34	12.42 ± 3.76	0.9243
		*p* ^∗∗^	0.619	**0.0016**	
	Perceived barriers	Experimental	27.14 ± 7.29	29.47 ± 5.68	**< 0.0001**
		Control	27.49 ± 6.88	29.47 ± 5.68	0.1527
		*p* ^∗∗^	0.820	**0.0144**	
	Perceived confidence/self‐efficacy of BSE	Experimental	28.09 ± 7.17	37.14 ± 5.94	**< 0.0001**
		Control	26.91 ± 7.83	28.30 ± 6.24	0.3507
		*p* ^∗∗^	0.466	**0.001**	
	Perceived health motivation	Experimental	21.84 ± 6.02	23.47 ± 6.88	**< 0.0001**
		Control	20.51 ± 6.07	23.47 ± 6.88	0.05
		*p* ^∗∗^	0.312	**0.0018**	

*Note:* Mean ± SD, Mean ± standard deviation. Scores were significant at a 0.05 level.

^∗^ *T*‐test independent groups.

^∗∗^ *T*‐test dependent groups.

When the intragroup scale scores of the experimental group were compared after the intervention, a significant difference was found in the subdimensions of perceived susceptibility, perceived BSE benefits, perceived barriers, perceived confidence/self‐efficacy of BSE, and perceived health motivation (*p* > 0.05) (Table 4).

## 4. Discussion

This study is the first to evaluate the effect of health education intervention, conducted among female university students in Somalia under the guidance of the HBM, on their breast cancer and early detection knowledge, BSE awareness, and related behaviors. The findings demonstrate that intervention designed in accordance with the HBM led to positive changes in participants’ health beliefs and improved their knowledge levels regarding breast cancer and early detection, as well as their BSE awareness and practice.

BSE and breast cancer awareness play a critical role in the early detection of breast abnormalities, particularly in low‐ and middle‐income countries where access to screening and diagnostic facilities is limited [33, 34].

Before the intervention, both groups exhibited comparable levels of knowledge and awareness; however, after the training, the experimental group showed remarkable gains in BSE knowledge, perceived benefits, self‐efficacy, and practice frequency. Following the intervention, participants in the experimental group demonstrated a significant increase in their knowledge of breast cancer, early diagnosis methods, and BSE awareness and practice. These results are consistent with studies among female university students in Ethiopia, Egypt, and Türkiye, which found that HBM‐based or structured educational programs enhanced breast cancer knowledge and regular BSE performance [35–37]. However, some studies have reported differing results, emphasizing that increased knowledge does not always translate into consistent behavioral change. For example, Al‐Sharbatti et al. [38] found that although awareness levels improved after breast health education among female students in the United Arab Emirates, BSE practice rates did not increase significantly. Similarly, Alotaibi et al. [39] observed that among Saudi nursing students, while breast cancer knowledge improved postintervention, many participants still expressed uncertainty or reluctance to perform BSE regularly [38, 39]. These results may suggest that culturally adapted and interactive educational interventions can effectively promote lasting improvements in breast cancer awareness and BSE practices among young women in low‐resource settings like Somalia.

According to the HBM, individuals’ engagement in preventive health behaviors depends on their perceptions of susceptibility, severity, benefits, and self‐efficacy related to a specific health condition [40, 41]. Perceived susceptibility refers to an individual’s belief about the likelihood of being affected by a certain disease. When a woman perceives herself to be at high risk for breast cancer, her sense of susceptibility increases. In our study, a significant difference was found in the experimental group in terms of the susceptibility subscale after the intervention, while no significant difference was observed between the groups. In line with our findings, some studies have also reported that participants’ perceived susceptibility scores increased following the intervention [42, 43]. However, in contrast to these findings, some studies have reported that participants’ perceived susceptibility scores did not show any significant change following the intervention [44, 45]. In our study, the fact that participants in the experimental group perceived themselves as more susceptible to the disease after the training suggests that the intervention was successful in raising awareness and promoting self‐protective health behaviors.

Perceived seriousness refers to the individual’s interpretation of the potential adverse consequences of a disease and reflects a woman’s level of acceptance and awareness regarding the possible changes that may occur in the event of developing breast cancer. In our study, no significant differences were observed within or between groups regarding the perceived seriousness subscale. Consistent with our findings, previous studies have also reported no statistically significant difference in perceived seriousness scores between women who performed BSE and those who did not [45, 46]. However, other studies evaluating the effect of breast cancer awareness education have reported a significant difference between groups in the posttest measurements [42, 44, 45]. Our study results suggest that Somali women’s fatalistic attitudes toward future events, influenced by their religious beliefs, combined with the relatively young average age of the participants, may have led them to perceive breast cancer as a condition distant from themselves.

Perceived benefits refer to an individual’s beliefs about the positive outcomes of adopting preventive and early detection behaviors for breast cancer, which play an important role in shaping such health‐promoting actions. In our study, a significant difference was observed not only within the experimental group before and after the intervention but also between the experimental and control groups following the education. Several studies have reported that participants who received information about BSE evaluated its advantages significantly higher, and these findings are consistent with the results of the present study [24, 42–44, 47, 48]. However, Kıssal and Kartal reported in their study that there was no significant change in perceived benefits [45]. This finding may indicate that the significant improvement in perceived benefits observed in our study could be attributed to the comprehensive content of the health education program and the interactive approach used during the intervention, which effectively enhanced participants’ understanding and motivation toward preventive behaviors.

According to the HBM, the internal and external difficulties that women experience during breast cancer screenings and BSE practices are defined as perceived barriers. The findings of the present study indicate that perceived barriers increased in both groups following the breast cancer and BSE education. After the intervention, it was anticipated that the experimental group’s perceived barriers would decline. However, unlike our study, other studies have shown a decrease in the perception of obstacles after the intervention compared to the beginning of the intervention [24, 44, 45, 48]. However, some studies have reported that although there was a decrease after the intervention, the difference was not statistically significant [42, 45]. The results of this study suggest that the increase in perceived barriers may be attributed to the participants’ young average age, the fact that this was the first study conducted at a university in Mogadishu, Somalia, and the low levels of perceived seriousness toward breast cancer among the participants, all of which may have contributed to the elevated perception of barriers.

Self‐efficacy (confidence perception) refers to an individual’s perceived competence in performing health‐related behaviors and plays a critical role in both initiating and maintaining behavioral change. In our study, both within‐group and between‐group analyses revealed that the female students in the experimental group demonstrated higher self‐efficacy compared to those in the control group. This finding is consistent with the results of previous studies [24, 42–44, 47, 49, 50]. In contrast, the study by Kıssal and Kartal did not report a significant difference in self‐efficacy levels and suggested that this outcome may have been related to the participants’ limited opportunities to practice BSE [45]. The increase in self‐efficacy observed in our study suggests that the intervention may have strengthened young Somali women’s confidence and perceived competence in performing BSE. This finding also indicates that the educational program may have played an effective role in enhancing participants’ awareness of breast cancer.

Health motivation refers to an individual’s general willingness and conscious effort to develop a particular health behavior. In this study, students’ observation of BSE being performed on a model breast, watching an educational video, receiving information through a PowerPoint presentation, being followed and supported by researchers for 3 months, and receiving individual training may have contributed to the positive impact on participants’ health motivation after the intervention. Similarly, many studies in the literature have demonstrated that such interventions positively influence women’s perceived health motivation scores [44, 47, 50]. On the other hand, four different studies [24, 45, 51] reported that the intervention had no significant effect on participants’ perceived health motivation scores.

## 5. Limitations

There were certain limitations to this study. First, since the research was conducted among young, educated women and limited to a single university, the findings cannot be generalized to all young women in Mogadishu, Somalia. It is recommended that future intervention studies include women from lower socioeconomic backgrounds residing in different neighborhoods of Mogadishu to enhance representativeness. Additionally, as all data in this study were self‐reported and no objective measures were employed to assess participants, this constitutes another limitation of the research.

## 6. Conclusions

This randomized controlled study demonstrated that HBM‐based breast health education significantly improved Somali female university students’ knowledge, awareness, and beliefs regarding breast cancer and BSE. Moreover, it strengthened their self‐efficacy and motivation to engage in preventive health behaviors. The findings highlight the feasibility and effectiveness of theory‐driven educational interventions in resource‐limited settings where access to mammography and early detection services is scarce. Given the cultural context and health system constraints in Somalia, integrating structured, culturally adapted breast health education into university and community programs may serve as a cost‐effective strategy to enhance women’s participation in early detection practices. Future large‐scale, multicenter studies are warranted to validate these findings and to explore long‐term behavioral outcomes. Ultimately, BSE‐focused education represents not merely an awareness initiative but a crucial, evidence‐informed public health intervention capable of empowering women and mitigating breast cancer–related morbidity and mortality in low‐resource contexts.

## Author Contributions

(i) Conception and design: all authors; (ii) administrative support: Şeyma Zehra Altunkurek; (iii) provision of study materials or patients: Şeyma Zehra Altunkurek and Samira Hassan Mohamed; (iv) collection and assembly of data: Şeyma Zehra Altunkurek and Samira Hassan Mohamed; (v) data analysis and interpretation: Şeyma Zehra Altunkurek and Eylül Yeşilyurt; (vi) manuscript writing: all authors.

## Funding

This research received no external funding.

## Disclosure

All authors approved the final version of the manuscript.

## Conflicts of Interest

The authors declare no conflicts of interest.

## Data Availability

The data used to support the findings of this study are available from the corresponding author upon request.
